# Test for Quinia Sulphate

**Published:** 1880-02

**Authors:** 


Test for Quinia Sulphate.—The test is based on two facts. 1. Water at 50-60° C. dissolve quinia sulphate but sparingly, while the other sulphates are readily dissolved. 2. When the cold solution is oversaturated with ammonia and then agitated with ether, just sufficient to dissolve the quinia present, this quantity will not suffice to take up the other alkaloids when exceeding a certain percentage.

First construct a *chininometer* by taking a test tube 120 mm. high and 10–12 mm. in diameter, which mark in such a manner that a line will indicate 5 cubic centimetres (a-b,) while another line above this shall indicate one cem. more (b–c.) Place 0.5 gramme of quinia sulphate in a test tube, pour over it 10 ccm. of hot water (50–60° C.,) shake well and allow to cool. After ten minutes pour into a small filter, (diam. 60 mm.,) collect in the chininometer 5 cem. of the filtrate, a–b; to the latter add 1 ccm. of ether, b–c, and 5 drops of ammonia sp. gr. 0.960; insert a well fitting cork into the chininometer, shake gently several times, then put aside for two hours. On examining the supernatant ether by means of a lens, no crystals should be observed suspended in the liquid, in which case the quinia salt is sufficiently pure.

If not more than 0.25 pc. cinchonia sul., or 0.5 pc. quinidia sul., 1.0 pc. cinchonidia sul. or 1.0 pc. homocinchonia sul. are present, they will not be indicated.—*Arch. d. Pharm.*

